# Exploitation of Cellular Cytoskeletons and Signaling Pathways for Cell Entry by Kaposi’s Sarcoma-Associated Herpesvirus and the Closely Related Rhesus Rhadinovirus 

**DOI:** 10.3390/pathogens1020102

**Published:** 2012-10-22

**Authors:** Wei Zhang, Shou-Jiang Gao

**Affiliations:** Department of Molecular Microbiology and Immunology, Keck School of Medicine, University of Southern California, Los Angeles, CA90033, USA; E-Mail: zhangw3048@gmail.com

**Keywords:** Kaposi’s Sarcoma-Associated Herpesvirus (KSHV), Rhesus Rhadinovirus (RRV), Virus Entry, Endocytosis, Actin, Microtubule, Integrin, Cellular Signaling, Ubiquitination

## Abstract

As obligate intracellular pathogens, viruses depend on the host cell machinery to complete their life cycle. Kaposi’s sarcoma-associated herpes virus (KSHV) is an oncogenicvirus causally linked to the development of Kaposi’s sarcoma and several other lymphoproliferative malignancies. KSHV entry into cells is tightly regulated by diverse viral and cellular factors. In particular, KSHV actively engages cellular integrins and ubiquitination pathways for successful infection. Emerging evidence suggests that KSHV hijacks both actin and microtubule cytoskeletons at different phases during entry into cells. Here, we review recent findings on the early events during primary infection of KSHV and its closely related primate homolog rhesus rhadinovirus with highlights on the regulation of cellular cytoskeletons and signaling pathways that are important for this phase of virus life cycle.

## 1. Introduction

Entry into cells is the first step in a successful viral infection [1]. A myriad of dynamic interactions between the virus and cell occurs during this complex process [2]. It is not surprising that viruses have evolved diverse strategies to usurp cellular structures and functions to ensure their successful infections. Kaposi’s sarcoma-associated herpesvirus (KSHV) is a gammaherpesvirus associated with several human malignancies primarily found in immunocompromised subjects including Kaposi’s sarcoma (KS), primary effusion lymphoma (PEL), and a subset of multicentric Castleman’s disease (MCD) [3]. Studies in the last decade have illustrated the pathways of KSHV entry into different cells, and identified viral and cellular factors that regulate this process. Some of these events have been discussed in previous reviews [4,5]. Recent advances indicate that cellular cytoskeletons and their associated signaling pathways regulate different phases of the viral life cycle, including those of herpesviruses [6,7,8]. 

Cytoskeletons are essential cellular components, which are involved in the formation of cellular structures, cargo transport and many other cellular functions [9]. Viruses subvert cytoskeleton barriers and dynamics to promote their internalization through plasma membrane fusion or endocytosis [6,7,8]. There is emerging evidence that KSHV actively modulates and engages cytoskeletons during its entry into cells, and these dynamic virus–cell interactions are essential for KSHV infection. In this review, we first outline the nature of cellular cytoskeletons and early events of KSHV entry including a newly identified cellular receptor ephrin receptor tyrosine kinase A2 (EphA2) [10]. We then focus on the role of cellular cytoskeletons, particularly actin, in the early events of KSHV infection, and discuss the latest updates on the involvement of cellular cytoskeletons and related signaling pathways in these processes. Finally, we make comparison of the early events of infection of KSHV with those of other viruses, particularly rhesus rhadinovirus (RRV), a KSHV closely related primate gammaherpesvirus.

## 2. Cytoskeletons

Cytoskeletons are intracellular fiber networks that fill the space between organelles within the cytoplasm. The cytoskeleton not only serves as the structure to cells but also plays important roles in many cellular functions, including mechanical support, cell division and intracellular transport [9,11,12]. Eukaryotic cytoskeletal filaments are composed of microtubules, actin filaments (also known as microfilaments), and intermediate filaments. These highly dynamic components, which constantly undergo cycles of polymerization/depolymerization, have different sizes and shapes, and contribute to various aspects of cellular functions [11,13]. 

### 2.1. Microtubules

Microtubules are hollow cylinders composed of α- and β-tubulin heterodimers [14]. Microtubules facilitate intracellular transport of vesicles, organelles and chromosomes. The relative stable microtubule “minus-end” is tethered to the microtubule organization center (MTOC) primarily composed of another type of tubulin, γ-tubulin, while the highly dynamic microtubule “plus-end” is anchored at cell membrane [15]. The function and dynamics of cellular microtubules are regulated by Rho GTPases and posttranslational modifications of tubulin subunits [16]. By switching between GTP-bound and GDP-bound form, Rho GTPases transduce diverse signals from cell membrane receptors to cytoskeletons [13,17,18]. The activities of Rho GTPases are mainly regulated by GTPase activating proteins (GAPs) and guanine nucleotide-exchange factors (GEFs) [19]. The best characterized Rho GTPases are RhoA, Cdc42 and Rac1 [20]. In addition to GTPases, microtubules are also regulated by posttranslational modifications, such as phosphorylation, acetylation and polyglycylation [21,22]. Although acetylation of tubulin subunits is involved in cell motility, the functions of these posttranslational modifications of tubulin subunits are not well understood [23,24]. Dynein and kinesin are two motor proteins responsible for the transport of cargos along microtubules. Both motors are ATPases but have distinct enzymatic activities and directional functions [25,26,27,28]. Transport from cell membrane to MTOC is mediated by dynein while kinesin moves cargos from minus-end toward plus-end. Many viruses rely on microtubules for their directional transport in cytoplasm [29,30,31,32,33,34,35,36,37].

### 2.2. Actin Filaments

Actin is one of the most abundant proteins in eukaryotic cells [38]. Actin is highly conserved across species and involved in many cellular functions, including maintenance of cell structures, cell motility, cytokinesis, and movement of cargos [39]. Actin filament (F-actin), which consists of two subunits of monomeric globular actin (G-actin), displays polarized plus (barbed) and minus (pointed) ends [40]. The actin plus end, which generally faces plasma membrane, grows rapidly to elongate the actin filament, while the minus end is relatively stable [39,40]. The continuous and reversible transition between G-actin and F-actin, which is regulated by Rho GTPases, sustains the dynamics of actin cytoskeletons [17].

Activation of specific GTPases, which leads to the activation of actin induction factors including Rho-associated kinases (ROCKs), formins, Wiskott-Aldrich syndrome protein (WASP), WASP-family verprolin homologues (WAVEs), actin-related protein 2/3 complex (Arp2/3) and other actin-binding proteins (ABPs), promotes actin polymerization and formation of actin structures such as filopodia, lamellopodia, and stress fibers [41]. RhoA regulates the formation of stress fibers while the reorganization of filopodia and lamellopodia requires Rac1 and Cdc42, respectively [41]. It has been reported that serum response factor (SRF) and its cofactor myocardin-related transcription factors (MRTFs) complex regulate the actin cytoskeleton remodeling [42]. In resting cells, G-actin sequesters MRTFs into the cytoplasm, resulting in the downregulation of SRF-targeting genes. Upon stimulation, Rho GTPases promote actin assembly. The reduced level of cytoplasmic G-actin leads to the release of MRTFs. Translocation of MRTFs into nucleus permits their interactions with SRF, resulting in the induction of cytoskeletal genes. These target genes, including actin and actin modulators, further enhance the polymerization of actin in the cytoplasm.

The movement of cargo along actin cytoskeletons is assisted by the myosin motor family. Myosin family members are ATPases, which provide energy for generating force and movement by hydrolyzing ATP [43,44]. All myosins bind to actin through their N-terminal motor domain and exert their cargo binding specificities via their diverse tail regions [45]. Most myosins move cargos in the direction of actin plus-end, but myosin VI and other members walk towards minus-end [46].

Numerous viruses have evolved mechanisms to disrupt or hijack actin for their successful infection [47,48,49]. KSHV also hijacks actin cytoskeletons for its entry and trafficking, which will be discussed in the following sections.

### 2.3. Intermediate Filaments

The third group of cytoskeletons is intermediate filaments, which are abundant in cytoplasm and nuclear membrane, and are essential parts for cell integrity [50]. Intermediate filaments are coiled-coil dimers composed of a variety of different fibrous proteins. Intermediate filaments are considered as the most stable and firm element of the cytoskeletons [51]. Therefore, it is not surprising that they are abundant in strong organizations such as hair and fingernails [52]. However, intermediate filaments are not responsible for cargo transport in mammalian cells because of their lack of polarity and associated motor proteins [53].

## 3. Kaposi’s Sarcoma-Associated Herpesvirus and Rhesus Rhadinovirus

Herpesvirus virions have four structural components. The relatively large double-stranded, linear DNA genomes are enclosed within icosahedral capsids of approximately 100 nm in diameter [54]. The nucleocapsids are wrapped with lipid bilayer envelopes containing viral glycoproteins. The amorphous space between the envelopes and nucleocapsids is filled by teguments, most of which have unknown functions [55,56]. 

Based on biological properties and sequence similarities, the herpesviruses family is divided into three subfamilies: the alphaherpesvirus, betaherpesvirus, and gammaherpesvirus [56,57]. Human gammaherpesviruses include Epstein-Barr virus (EBV) and KSHV, which have the narrowest cell and host range among all eight human herpesviruses identified so far [58,59].

KSHV, a gamma-2 herpesvirus (rhadinovirus), is one of the seven human tumor viruses identified so far [60]. KSHV has latent and lytic replication phases [61]. KSHV establishes persistent latent infection in the host following primary infection. During latency, the viral genome is maintained as multiple copies of circular episome in host cell nucleus, which replicates and persists with expression of a handful of latent genes. The viral episome can be reactivated into lytic replication, resulting in the expression of cascade of viral lytic genes, replication of linear viral genome, and production of infectious virions [61]. Full viral lytic replication often results in cell death. 

KSHV has a large genome encoding about 90 open-reading-frames (ORFs) and a cluster of 12 precursor microRNAs [62]. In addition to structural proteins and proteins involved in viral replication, KSHV encodes a number of proteins that regulate diverse cellular functions, including gene transcription, immune response, apoptosis, and cell cycle [3,62]. In immunocompetent hosts, infection by KSHV is generally asymptomatic; however, KSHV is associated with KS, PEL and MCD in immunocompromised subjects [63,64]. Of note, KS is the most common neoplasm in HIV-infected subjects [65].

RRV is closely related to KSHV [66]. Two RRV isolates (RRV 26–95 and RRV 17577) were independently identified [66,67]. Analyses of DNA sequences indicate that the RRV genome is aligned with, and shares a high degree of sequence similarity to KSHV [66,67]. In culture, RRV replicates more efficiently than KSHV. Furthermore, RRV infects rhesus macaques and induces MCD and non-Hodgkin’s lymphoma-like diseases in immunodeficient rhesus macaques, which is similar to KSHV-associated malignancies in individuals coinfected by HIV and KSHV [68,69]. Thus, there are attempts to develop an infection model of RRV for KSHV study [70]. 

## 4. Early Events in KSHV and RRV Infection

During primary infection, herpesviruses deliver their DNA into a nucleus through a series of well-organized entry events, involving many viral and cellular factors [56]. To gain access to the cell interior, herpesviruses attach and bind to specific entry receptor(s) through viral glycoproteins. The interactions of viral glycoproteins with their cellular receptors dictate subsequent internalization, membrane fusion and trafficking of the virus. Herpesviruses subvert diverse cellular pathways to facilitate their entry; however, it is often cell type-dependent [56,71]. Herpesviruses use both endocytosis and plasma membrane fusion to enter the cells. Following internalization and fusion, the nucleocapsids or nucleocapsids-containing vesicles are transported to a perinuclear space for the delivery of viral DNA into nucleus. Both actin and microtubule cytoskeletons are involved in multiple steps in herpesvirus entry and trafficking, and they may cooperate with each other to deliver viral genomes [6].

Similar to other herpesviruses, both KSHV and RRV use receptor-mediated endocytic pathway, and hijack cytoskeletons for their entry and trafficking. In the following sections, we will discuss the critical steps including attachment, receptor binding, internalization, fusion, and trafficking during KSHV and RRV entry of cells. We will also compare their behaviors with other viruses, particularly herpesviruses. The cellular pathways and factors that are involved in KSHV and RRV entry are summarized in Table 1.

**Table 1:** Cellular factors and pathways involved in early events of KSHV and RRV infection in different target cells.

Virus	Cell type	Attachment receptor	Binding receptor	Entry pathway	Regulation by cytoskeleton	Reference
KSHV	HUVEC	Heparan sulfate-gH/gL	EphA2-gH/gL	Clathrin-mediated endocytosis	Actin	[10,92,136,140]
KSHV	HMVEC-d	Heparan sulfate-gH/gL	α3β1-gB, EphA2-gH/gL	Macropinocytosis	Actin and microtubule	[92,94,111,125,137,144]
KSHV	HFF	Heparan sulfate-gB, gpK8.1A, gH	α3β1-gB, αVβ3, αVβ5	Clathrin-mediated endocytosis	Microtubule	[91,93,111,135]
KSHV	B cells	Heparan sulfate-gB, gpK8.1A	DC-SIGN	Endocytosis	Unknown	[104,105,106]
KSHV	THP-1 monocytes	Unknown	DC-SIGN	Clathrin- and caveolae-mediated endocytosis	Unknown	[104,105,106]
KSHV	293T cells	Heparan sulfate-gB, gpK8.1A	Unknown	pH-dependent, non-clathrin-, non- caveolae-dependent pathway	Unknown	[91,94,116]
KSHV	Non-permissive cells transfected with xCT	Unknown	xCT	Membrane fusion	Unknown	[129]
KSHV	HT1080 fibrosarcoma	Unknown	αVβ3-gB	Unknown	Unknown	[118]
RRV	Rhesus fibroblast	Heparan sulfate-gB	Unknown	Clathrin-mediated endocytosis	Microtubule	[96,141]

### 4.1. Viral Glycoproteins

Several herpesvirus glycoproteins play important roles in different stages of primary infection, including attachment, binding, fusion, and trafficking [72]. Herpesvirus glycoprotein B (gB) usually presents at the surface of virion envelope as a homodimer, and mediates attachment, binding and fusion [56,72]. Glycoprotein L (gL) and glycoprotein H (gH) form heterodimers that mediate fusion, and gL functions as a chaperon to assist in the proper folding, process and transport of gH [56,73]. Transient expression of KSHV gB, gH and gL is sufficient to trigger cell–cell fusion, suggesting that these three glycoproteins are members of the KSHV core fusion machinery [74]. KSHV encodes several unique glycoproteins including glycoprotein K8.1A (gpK8.1A) and glycoprotein K8.1B (gpK8.1B) that do not have any sequence homology with proteins of other human herpesviruses [75]. gpK8.1A and gpK8.1B are derived from alternative splicing of the same transcript, and gpK8.1A is the predominant one [76].

Several homologs of RRV glycoproteins including gB, gH and gL have been identified [66,67]. Although RRV gB shows high sequence similarity and identity with KSHV gB, the function of RRV gB has not yet been examined. RRV gH and gL are virion proteins, and their expression is enhanced by codon optimization and expression of ORF57 [77,78]. Examination of RRV glycoproteins from different isolates identified two discrete groups of gH, gL, and gB sequences, but the other four glycoproteins showed minimal divergences [67,79,80]. Because glycoproteins of gammaherpesviruses are involved in attachment, receptor binding and membrane fusion, these sequence variations suggest possible differential entry pathways and receptor usages by these isolates during RRV primary infection of different cell types.

The cell type specific entry pathway of herpesviruses is best illustrated by the fact that EBV entry into different cell lines requires distinct glycoprotein complex [81,82]. EBV glycoproteins gH, gL, and gp42 form a tripartite complex, which is required for entry of B cells while a bipartite complex that contains only gH and gL is used for infection of epithelial cells [81].

### 4.2. Attachment

Heparan sulfate, a sulfated polysaccharide, is ubiquitously expressed on the surfaces and in the extracellular matrix of cells [83,84]. By interacting with viral structural components, heparan sulfate and its derivatives serve as attachment factors or entry receptors for many types of viruses, including herpesviruses [2,56,85,86,87,88,89]. Heparan sulfate is also involved in KSHV attachment mediated by gB, gH, and gpK8.1A [90,91,92,93,94]. However, not all of these glycoproteins are required for KSHV entry, and they can compensate each other for attachment. For example, gpK8.1 is not required for KSHV entry in 293 cells albeit it is involved in attachment through interaction with heparan sulfate [95]. RRV infectivity is also inhibited by heparin, a molecule with structure similar to heparan sulfate, in rhesus fibroblasts (RFs) [96]. Based on the putative heparan binding domain on RRV gB, it is highly possible that RRV gB interacts with heparan sulfate for the initial attachment.

### 4.3. Receptor Binding and Fusion

Following attachment, herpesviruses bind to specific entry receptors, and activate downstream signaling pathways, some of which, in return, modulate the virus entry and trafficking pathway in a series of programmed events [56]. The virus entry pathway is mainly determined by the interaction of the viral glycoprotein(s) with the cellular receptor(s), which triggers the fusion of the viral envelope with plasma membrane or membrane of intracellular vesicles following endocytosis [97]. An example is the retrovirus avian sarcoma and leukosis virus (ASLV) envelope glycoprotein (EnvA), which binds to cell membrane receptor TVA for infection. Binding of ASLV to two isoforms of TVA, the transmembrane receptor TVA950 and the glycophosphatidylinositol (GPI)-anchored receptor TVA800, leads to variable entry and fusion kinetics [98]. Unlike TVA950, TVA800 lacks the transmembrane domain, suggesting that these two receptors may activate different signaling pathways leading to distinct entry kinetics. Herpesviruses use several receptors for successful infection in different cell types. Multiple receptors including DC-SIGN, integrins, EphA2 and xCT are involved in KSHV entry and fusion, which might result in the activation of distinct signaling pathways and entry kinetics depending on the cell types.

#### 4.3.1. DC-SIGN

Dendritic cell-specific intercellular adhesion molecule-3-grabbing non-integrin (DC-SIGN) serves as an attachment factor or entry receptor for several viruses [99,100,101,102,103]. DC-SIGN facilitates KSHV entry in human acute monocytic leukemia cell line THP-1, activated primary B lymphocytes, myeloid dendritic cells, and macrophages [104,105,106]. Although B cells are the main reservoirs of KSHV *in vivo, *they are usually refractory to KSHV infection *in vitro*, which may be due to the low cell surface expression of heparan sulfate [107]. Activated B lymphocytes, which have a higher DC-SIGN expression level, are more accessible for KSHV infection than resting B cells [105]. Transfection of DC-SIGN into cells that were resistant to KSHV infection also converted them to permissive cells. Furthermore, blocking DC-SIGN in THP-1 cells reduced KSHV entry without affecting virus binding, suggesting that DC-SIGN may serve as the KSHV receptor in these cells [104]. These results are highly relevant to KSHV-associated PEL and MCD. However, it is unclear whether DC-SIGN is expressed in endothelial cells and KS tumors. Thus, the role of DC-SIGN in KSHV entry in relation to KS development remains to be determined. 

#### 4.3.2. Integrins

Integrins are a family of receptors that mediate cell migration, adhesion and many other cellular functions by integrating extracellular stimuli with the intracellular cytoskeletons [108]. Integrins are widely expressed in different types of cells including endothelial cells and B-cells. Integrins are composed of α and β transmembrane glycoprotein subunits. The diverse combinations of α/β integrins provide distinct binding specificities and signaling properties [109]. Integrins serve as receptors for many viruses [2,110]. KSHV is the first herpesvirus demonstrated to use integrins as the cellular receptors for infection [111]. RGD motif (arginine-glycine-aspartic acid) in multiple ligands is a recognition motif that mediates interaction with different integrins, including αV integrins, and α3β1, α5β1, α8β1 and αIIbβ3 integrins [112,113]. Integrin α3β1 interacts with the RGD motif of KSHV gB and serves as an entry receptor in human foreskin fibroblasts (HFF) and human dermal microvascular endothelial cells (HMVEC-d) [111]. The specificity of integrin α3β1 in mediating KSHV infection was demonstrated by using anti-integrin antibodies and soluble integrins. Furthermore, transfection of nonpermissive CHO cells with human α3 rendered them susceptible to KSHV infection. KSHV gB mediates cell adhesion through the interaction of its RGD motif on the extracellular domain with cell surface integrins [114]. However, subsequent studies demonstrated that αVβ3 and αVβ5 were as important as α3β1 for KSHV infection in HFF and HMVEC-d [115].

Inoue *et al* have reported that KSHV entry in 293T cells is α3β1 independent [116]. KSHV infection is insensitive to the treatment of RGD-motif-containing molecules. Pretreating KSHV inoculums with either integrin α3β1 or α5β1 does not significantly reduce KSHV infectivity, indicating that other molecules may serve as the KSHV entry receptor in 293T cells [116]. KSHV infection is also independent of integrin α3β1 in DC-SIGN-expressing cells [106]. Furthermore, there is a lack of correlation between KSHV permissive cell lines and the expression level of integrin α3β1, suggesting α3β1 is not the only receptor for KSHV infection [117].

KSHV entry into human fibrosarcoma HT1080 cells depends on integrin αVβ3 but not α3β1 [118]. KSHV gB has sequence similarity with ligands of integrin αVβ3. The RGD peptide of gB mediates adhesion of αVβ3-CHO but not α3β1-CHO cells. Anti-αVβ3 antibodies but not anti-α3β1 antibodies specifically block HT1080 adhesion to the gB RGD peptide. KSHV infection in HT1080 is blocked by an anti-αVβ3 antibody or a cyclic RGD peptide [118]. Furthermore, αVβ3 interacts with both the gB RGD peptide and KSHV virion. However, for coupling purposes, the authors used a gB RGD peptide with additional “GCG” amino acids at the C-terminal. The amino acids flanking RGD motifs have the potential to influence receptor specificity [119]. Although “GCG” are several amino acids away from RGD motif, it remains important to rule out the effect of “GCG” on the recognition specificity.

#### 4.3.3. EphA2

The ephrin subfamily is the largest in the receptor tyrosine kinases (RTK) superfamily with at least 16 receptors [120]. EphA2 is a receptor for its membrane-bound ligands, the Ephrin-A family members. The interactions of Ephrins and EphA receptors lead to bidirectional signaling into both cells, and are important for regulating cell adhesion and cell migration. EphA2 is one of the most commonly dysregulated Eph kinases receptor in human cancers [120,121,122]. EphA2 is expressed in both endothelium and lymphoid tissues [123]. Hahn *et al* have demonstrated that EphA2 serves as a KSHV entry receptor and directly interacts with gH/gL complex [10]. Disruption of EphA2 and gH/gL interaction impairs KSHV infection. EphA2 is expressed in KS tumors, and its expression level is positively correlated with KSHV infection *in vitro*. Furthermore, KSHV infection is reduced in mouse endothelial cells with both *Epha2* alleles knocked-out. However, the exact stage(s) of KSHV entry mediated by EphA2 and gH/gL interaction remains to be further explored. An earlier study has shown that KSHV infectivity is neutralized by both anti-gH and anti-gL antibodies in a post-binding step [124]. A separate study has also shown that EphA2 is essential for macropinocytosis and trafficking of KSHV but not attachment [125]. EphA2 is associated with KSHV as well as integrin α3β1 and αVβ3 early during infection, suggesting one role of EphA2 as a scaffold protein for recruiting entry and signaling complex for a successful infection. A recent study has shown that Ephrin-B2 mediates KSHV induced-angiogenesis through EZH2 (Enhancer of Zeste homolog 2) [126]. It remains to be determined if gH/gL-EphA2 signaling in KSHV primary infection could also contribute to KSHV-induced angiogenesis.

#### 4.3.4. xCT

Human cystine/glutamate exchange transporter system x^–^_c_ (xCT) is a multipass membrane protein that protects cells against stress by transporting amino acids [127,128]. It is widely expressed in different cell types. Kaleeba *et al* have demonstrated that xCT mediates cell fusion in the presence of KSHV glycoproteins [129]. Expression of xCT on KSHV nonpermissive cells renders them susceptible to KSHV infection. Furthermore, xCT neutralizing antibodies block cell fusion and KSHV infection into host cells. However, virus entry was measured using a recombinant KSHV containing a GFP cassette at 7 days post infection. At this time point, the expression of GFP could be influenced by factors other than virus entry, and thus might not represent the accurate virus entry event. Furthermore, although cell fusion is widely used for virus entry study, it fails to mimic the virus–host interaction in the context of virus infection. A later study has demonstrated the importance of xCT in KSHV viral gene expression rather than entry in an unclear mechanism [115]. Therefore, it is possible that xCT functions as a cofactor/fusion receptor to assist KSHV entry or the interaction between KSHV and xCT might trigger downstream signaling events essential for viral gene expression.

In summary, although there are some controversial data on the functions of integrin α3β1 and xCT during KSHV entry into cells, certain integrins, EphA2, and DC-SIGN are likely genuine cellular receptors for KSHV entry in specific cell types.

#### 4.3.5. RRV Receptors and Fusion

The receptor(s) mediating RRV entry has not been identified. RRV gB shares high sequence homology with KSHV gB. It has been speculated that RRV might use integrins as receptors for infection. However, RRV interacts with neither integrin α3β1 nor αVβ3 [118]. Our unpublished data also suggests that integrins do not serve as entry receptors for RRV in RFs. 

### 4.4. Internalization

Herpesvirus internalization occurs through either pH-independent plasma membrane fusion or endocytosis, which usually requires a low pH [56,130]. Herpesviruses exploit various internalization pathways for successful infection in different cell types. For example, EBV infects most B cells through endocytosis, while in epithelial cells, EBV internalization occurs by plasma membrane fusion [81,131,132,133]. Interestingly, EBV entry into some B-lymphoblastoid cells such as Raji cells occurs through plasma membrane fusion [131,132,134]. 

KSHV infects most cell types through endocytic pathways. For example, KSHV enters HFF and human umbilical vein endothelial cells (HUVEC) via clathrin-mediated endocytosis [135,136]. The impaired KSHV entry in both cell types by inhibitors of endosomal acidification suggests a pH-dependent endocytic pathway. Electron microscopy revealed the presence of KSHV particles in the endocytic vesicles in HFF cells [135]. KSHV entry is sensitive to inhibitors for clathrin- but not caveolae-mediated endocytosis, demonstrating that clathrin-mediated endocytosis is the main pathway for KSHV infection in fibroblasts and endothelial cells [135,136]. Furthermore, KSHV particles are colocalized with markers of clathrin-mediated endocytosis during infection of HUVEC [136]. KSHV particles are also colocalized with early endosome antigen 1 (EEA1) and late endosome/lysosome marker LAMP-1, indicating that KSHV may fuse its envelope membrane with lysosome to release nucleocapsid [136]. Importantly, the inhibitors described in these studies are commonly used for investigating virus entry and endocytosis of other viruses. In these KSHV studies, cytotoxicity assays such as by propidium iodide staining did not reveal any toxicity under the experimental conditions [135,136]. Furthermore, a number of assays were used to demonstrate the specificity of the inhibitors.

KSHV infects HMVEC-d cells through macropinocytosis, another endocytic pathway [137]. Inhibitors of macropinocytosis but not other endocytic pathways reduced the expression of KSHV genes without affecting virus binding. Most but not all gpK8.1A+ KSHV particles were colocalized with a marker of macropinocytosis [137]. Orf65+ KSHV particles were colocalized with a late endosome/lysosome marker LAMP-1 but not an early endosome marker EEA1. This study suggests that KSHV infection in endothelial cells might occur through macropinocytosis, which contradicts our observation that KSHV infection in HUVEC is mainly through clathrin-mediated endocytosis [136]. Several explanations might account for these differences. First of all, only one inhibitor of each endocytic pathway and a low concentration of each inhibitor were used in this study [137]. In contrast, clathrin-mediated endocytosis and KSHV entry were strongly inhibited by two different inhibitors of clathrin-mediated endocytosis at higher but nontoxic concentrations without affecting other endocytic pathways [136]. Secondly, direct visualization of virus particles by fluorescence microscopy or electron microscopy is a better method to track virus entry than assays relying on gene expression [138]. Finally, viral glycoproteins and tegument proteins are not suitable for tracking virus entry and trafficking [56,139] while visualization of single viral particle by staining capsid protein Orf65 is a more direct and sensitive way to measure the early events during primary infection of gammaherpesviruses [96,136,140,141]. 

Studies in other cell types show that KSHV entry into B cells is related to DC-SIGN-mediated endocytosis [105]. KSHV enters THP-1 cells via clathrin- and caveolae-mediated endocytosis which requires the endosomal acidification but does not utilize macropinocytosis [104]. KSHV also infects 293T cells by a pH-dependent, non-clathrin- or caveolae-dependent endocytic pathway [116]. Therefore, KSHV entry into permissive cells is cell type-dependent, but clathrin-mediated endocytosis is the major pathway for most cell types.

RRV entry into RFs also occurs through clathrin-mediated endocytosis [96]. Inhibitors of endosomal acidification reduce RRV infectivity in RFs, indicating a pH sensitive route of RRV entry. Furthermore, inhibitors of clathrin-mediated endocytosis but not those of caveolae-mediated endocytosis reduce RRV infectivity in RFs, suggesting that clathrin-mediated endocytosis serves as the major pathway for RRV entry. RRV particles are colocalized with a maker of clathrin-mediated endocytosis, clathrin subunits and EEA1, but not that of caveolae-mediated endocytosis, indicating that RRV is internalized via a route involving clathrin-coated vesicle and early endosome. Consistent with these results, RRV entry is blocked by overexpression of a dominant-negative construct epidermal growth factor receptor pathway substrate 15 (EPS15) [96], which is an essential component of clathrin-coated pits [142,143]. Together these results show that RRV entry into RFs is mediated by clathrin-mediated endocytosis.

### 4.5. Trafficking

Following virus internalization, the nucleocapsids-containing vesicles or free nucleocapsids are transported in the cytoplasm toward nucleus. Free diffusion of molecules or organelles to specific cellular destination is a slow and inefficient process in the highly crowded cytoplasm [6,17,47,138]. Therefore, many viruses employ active, energy-consuming strategies for their transport. Cytoskeletons, especially microtubule and actin cytoskeletons, are involved in many steps, including, but not limited to, serving as super highways for the incoming virus particles to reach the nuclei, during the early stages of virus infection [47].

KSHV trafficking in endothelial cells depends on cytoskeletons [136]. KSHV primary infection remodels actin dynamics, and KSHV particles are associated with actin at different infection stages in HUVEC. Furthermore, disruption of actin dynamics reduces total number of KSHV particles reaching each nucleus. The incomplete inhibitory effect of actin-depolymerizing agent, and the colocalization of Orf65+ viral capsids with microtubules suggest that KSHV might also take advantages of the microtubule networks for nuclear targeting [136].

KSHV infection induces the polymerization of microtubules and the formation of thick bundles. Disruption of microtubule but not actin cytoskeletons strongly inhibits KSHV trafficking in HFF cells [144]. To move along the microtubules, virus particles interact with the microtubule motor protein dynein–dynactin complex to gain access to nucleus. Overexpression of dynamitin, one of the subunit of dynactin, impairs dynein-mediated retrograde microtubule transport [145]. KSHV trafficking is strongly reduced following treatment with an inhibitor of dynein indicating that a functional microtubule motor complex is required for KSHV infection [144]. However, it is unclear whether there is a direct interaction between the viral component and the dynein complex during trafficking. 

The role of microtubule networks in RRV trafficking in RFs has also been investigated [141]. Modulations of microtubules with either depolymerizing or stabilization drugs inhibit RRV nuclear trafficking, suggesting that both intact microtubules and microtubule dynamics are required for RRV trafficking to the perinuclear regions. Furthermore, RRV nuclear targeting is reduced by an inhibitor of the dynein motor or by overexpression of dynamitin, indicating that this process is dynein-dependent. Furthermore, RRV particles are colocalized with microtubules and the microtubule-based motor protein dynein. Together, these data demonstrate that both microtubule and dynein–dynactin complex are involved in the transport of incoming RRV particles to perinuclear regions during RRV primary infection.

## 5. Involvement of Cytoskeletons in Gammaherpesvirus Infection

### 5.1. The Role of Actin Cytoskeletons in Herpesvirus Attachment and Binding

Many viruses actively regulate actin cytoskeletons through a series of signaling events at different stages of their life cycle, particularly during *de novo* infection [17,47]. Adenovirus attaches to the GPI-anchored complete decay-accelerating factor (DAF) for the initial attachment, and reorganizes cytoskeletons by activating tyrosine kinase ABL and GTPases. The actin motor myosin II promotes adenovirus movement by regulating cell projections such as filopodia, a process called “virus surfing”, to reach the coxsackie and adenovirus receptor (CAR), which is located at tight junctions between epithelial cells, and thus hidden from the incoming virions [17,146]. Most herpesviruses, such as herpes simplex virus 1 (HSV-1), attach to cell membrane or filopodia through the interactions of viral glycoproteins with cell surface heparan sulfate [85]. This process is reversible but helps the herpesviruses to concentrate on cell surface. Herpesvirus surfing is also mediated by myosin in the filopodia or cortical actin. Actin drags the virion to search for receptors and to the site of entry on the cell membrane [17,147]. For example, HSV-1 modulates filopodia to promote the virion surfing along the cell membrane through Cdc42 activation, probably mediated by myosin [148]. A recent study demonstrates that nonmuscle myosin IIA serves as the HSV-1 receptor by interacting with gB, further suggesting a role of actin and myosin motor protein during the binding step of HSV-1 infection [149]. Because of the diverse attachment and binding receptors, gammaherpesviruses might also utilize cell membrane surfing as a mechanism to search for their entry receptor(s) and move to the region with high endocytic activities in some cell types.

### 5.2. The Role of Actin Cytoskeleton in Herpesvirus Internalization

Although the role of actin in plasma membrane fusion is still under debate, results of recent studies have indicated the involvement of actin during endocytosis of many virus families [7]. Actin plays an important role in HSV-1 internalization in some cell types. In human corneal fibroblasts and nectin-1-CHO cells, HSV-1 infection activates RhoA and Cdc42 through its receptor nectin-1, followed by cytoskeleton remodeling, which is required for HSV-1 endocytosis [150]. Similarly, HSV-1 activates Rac1 and Cdc42 to promote entry into MDCKII cells and keratinocytes, probably by modulating actin cytoskeletons [151,152]. However, HSV-1 entry into Vero cells, which occurs through pH-independent plasma membrane fusion, is actin-independent [153].

Actin cytoskeletons are also involved in EBV infection. Inhibition of actin remodeling decreases the efficiency of EBV entry into B cells but surprisingly enhances the internalization of EBV in epithelial cells [154]. EBV infects B cells through endocytosis while enters epithelial cells via plasma membrane fusion [81]. Therefore, the disruption of actin under cell membrane might promote the internalization and infection of EBV in epithelial cells. Human cytomegalovirus (HCMV) also disrupts actin during primary infection to enhance its infectivity [155].

KSHV modulates cytoskeletons and associated signaling pathways during entry. Integrins play an important role in the reorganization of actin cytoskeletons [42]. KSHV induces outside–in integrin signaling pathway through focal adhesion kinase (FAK) for successful infection in HFF [111,156]. FAK is localized at focal adhesion (FA) with other molecules, including Src and actin [157]. KSHV infection induces FAK-Src-PI3K pathway to activate RhoA and Cdc42, which prompts rapid polymerization of actin, leading to increased stress fiber and filopodia in HFF [158,159]. RhoA activates diaphanous-2 to modulate cytoskeleton dynamics and promotes KSHV infection [144]. Diaphanous-2 in turn activates Src, thus generating a positive feedback loop resulting in constitutive activation of Src, which is essential for KSHV infection [160]. Src also regulates the translocation of clathrin subunits to facilitate the formation of the clathrin-coated pits [161]. In addition, treatment with genistein, a tyrosine kinase inhibitor targeting FAK, Src and PI3K, strongly inhibits KSHV internalization, indicating that actin remodeling mediated by Rho GTPases is required for KSHV endocytosis in HFF [135,159].

The role of actin in KSHV entry and trafficking has also been studied in endothelial cells [136,137,144]. Actin-depolymerizing agent inhibits KSHV gene expression and prevents KSHV entry in HMVEC-d, suggesting the potential involvement of actin in endocytosis during KSHV entry into endothelial cells [137,144]. Similar to HFF, KSHV infection induces actin remodeling and formation of stress fiber, filopodia and lamellipodia in endothelial cells [136,137,158,159].

### 5.3. The Differential Role of Actin Cytoskeletons and Related Signaling in KSHV Entry through Clathrin-Mediated Endocytosis and Macropinocytosis

KSHV infects most cell types through clathrin-mediated endocytosis. The role of actin cytoskeletons in regulating clathrin-mediated endocytosis is known in yeast but it is less clear in mammalian cells [162]. In mammalian cells, the formation of a clathrin-coated vesicle requires the following steps: cell surface accumulation of clathrin-coated subunit; initial invagination of coated pit; elongation of coated pits to form a necked bud; scission of clathrin-coated vesicle from the neck; and the departure of clathrin-coated vesicle from plasma membrane [163,164]. Whether any of these stages are involved with actin in clathrin-mediated endocytosis of mammalian cells is still in debate [165]. Actin has been shown to form a “collar” structure along the neck of coated pits to elongate the bud, and facilitate the departure from plasma membrane after neck scission [12]. Thus, actin might be involved in the late stage of formation of clathrin-coated vesicles. Adenovirus has been shown to activate Rac and CDC42 through PI3K, and stimulate actin remodeling and the formation of lamellipodia to facilitate its endocytosis [49]. However, the exact stage of actin remodeling during clathrin-mediated endocytosis of KSHV is not known. Therefore, the role of FAK signaling in actin remodeling of KSHV endocytosis through individual Rho GTPase need to be further explored.

In mammalian cells, Cbl (Casitas B-lineage lymphoma), including c-Cbl, Cbl-b and Cbl-c, is a family of E3 ligases implicated in signal transduction [166]. During KSHV macropinocytosis in HMVEC-d cells, c-Cbl is phosphorylated, which is then associated with PI3K to promote the formation of bleb, one type of actin protrusion formed during macropinocytosis, at the cell membrane [167,168]. KSHV infection of HUVEC also activates c-Cbl, which is dependent on Src activation and is required for KSHV entry [140]. KSHV particles are localized with c-Cbl during entry of HUVEC. EphA2 also interacts with c-Cbl and myosin IIA to facilitate the formation of bleb, further suggesting the involvement of actin in KSHV macropinocytosis [169].

### 5.4. The Role of Cytoskeletons in Herpesvirus Endosomal Sorting and Trafficking

Cytoskeletons play a significant role in intracellular movement of viruses toward nucleus. Microtubules and the associated motor protein dynein have been shown to mediate intracellular trafficking of many viruses. For example, Adenovirus-2 and -5 attach to microtubule for trafficking [15]. KSHV infection of HFF activates RhoA and Rac1, and modulates the microtubule dynamics in part by acetylation, which is likely mediated by signaling pathways induced during virus entry [144]. Although dynein-dynactin complex is essential for retrograde movement of many herpesviruses along microtubules, no herpesvirus protein has been shown to interact with the dynein complex, indicating that herpesviruses may move within intracellular vesicles along microtubules to reach perinuclear region.

Of interest, actin has been shown to regulate herpesvirus intracellular transport. Treatment of HUVEC with inhibitors of Rho GTPases or actin regulators leads to the disruption of actin filaments and inhibition of KSHV entry and trafficking, suggesting the important role of Rho GTPases and proper regulation of actin assembly during KSHV primary infection [136]. The observation that KSHV particles are colocalized actin cytoskeletons and intracellular vesicles suggests that they might be transported within early endosome, multivesicular bodies (MVBs), late endosome and lysosome before membrane fusion [136]. The involvement of actin in endosomal sorting is supported by the observed colocalization of KSHV particles with transferrin, actin and endosome/lysosome markers [136]. Actin and its associated proteins have also been shown to provide mechanical force for endosomal maturation [170]. In fact, KSHV particles are colocalized with both actin and microtubule cytoskeletons during entry and trafficking [136]. Thus, actin and microtubule cytoskeletons could cooperate with each other to move virion-containing intracellular vesicles toward nucleus by promoting endosomal maturation and long-distance transport, respectively. Although membrane fusion of viruses may occur at distinct stages where fusion machinery is activated, fusion with late endosome or lysosome appears to have several advantages. Firstly, viruses could be protected in the intracellular vesicles to pass the crowded cytoplasm and to be efficiently transported to specific locations before their release from endosomes. Secondly, during endocytosis, the low pH of endosome/lysosome triggers the conformational changes of the fusion proteins, resulting in membrane fusion between the viral envelope and the intracellular vesicle membrane through stepwise confirmation changes. Lastly, for viruses that have a small genome, it provides a convenient way for their transport to the perinuclear spaces before fusion, avoiding the need to encode protein(s) required for interaction with the dynein–dynactin complex or other components of the transportation machinery.

## 6. Regulation of Gammaherpesvirus Infection by Cellular Signaling Pathways

Ubiquitination is involved in many important cellular pathways, including transport of molecules through endocytosis and signal transduction in cells [171]. Poly-ubiquitination usually mediates the degradation of targeted proteins by proteasome, while mono-ubiquitination alters their function and cellular distribution. For example, mono-ubiquitination of transmembrane proteins such as receptors triggers signal transduction to facilitate the internalization and endocytic sorting of proteins [172]. Treatment with proteasome inhibitors significantly reduces KSHV infection in HUVEC, and traps the incoming virus particles in early endosomes [140]. KSHV particles are colocalized with ubiquitin-binding proteins, and ubiquitination regulates the internalization of KSHV and integrin β1. KSHV entry and trafficking, as well as KSHV-induced ubiquitination of one of its receptors integrin β1, is sensitive to inhibitor of E1-activating enzyme [140]. As stated above, KSHV activates c-Cbl in HUVEC, and KSHV productive infection requires c-Cbl. These data suggest that ubiquitination and E3 ligase c-Cbl play important roles in KSHV entry and trafficking, including sorting of KSHV-containing endosomes. c-Cbl mediates the ubiquitination of KSHV receptors α3β1 and αVβ3 for productive infection [140,169].

KSHV modulates multiple signaling pathways during primary infection. KSHV activation of ERK, JNK, and p38 multiple mitogen-activated protein kinase (MAPK) pathways are essential for virus entry, expression of viral genes, and an early stage of productive viral replication [173,174,175]. While the mechanism by which MAPK pathways regulate KSHV entry is unclear, we have shown that they mediate the activation of activator protein 1 (AP-1) during KSHV entry, which is required for viral gene expression, genome replication, and productive replication [173,175]. Activation of AP-1 also leads to induction of several proinflammatory and angiogenic cytokines, including matrix metalloproteinase (MMP)-1, MMP-2, MMP-9, IL-6 and angiopoietin-2, which promote the progression of KSHV-induced malignancies, and possibly dissemination of infectious viruses *in vivo* [173,175,176,177]. Interestingly, KSHV entry induces VE-cadherin degradation and increases vascular permeability [178]. While increased vascular permeability and induction of MMPs might promote virus transmission, it is unclear whether they could enhance KSHV entry in the effector cells. Nevertheless, it has been shown that disruption of adherens junction liberates the entry receptor nectin-1, facilitating cell entry of HSV-1 and pseudorabies virus [179]. 

## 7. Conclusions

Successful virus infection requires the proper delivery of the viral genome into the infected cells in an orchestrated manner. Herpesvirus entry is a complicated cell type-dependent process involving viral glycoproteins, cellular factors, and diverse signaling pathways. Besides serving as transportation systems for the delivery of herpesvirus nucleocapsids to a nucleus during primary infection, accumulated evidence suggests that cytoskeletons, particularly actin, also play other important roles in different stages of herpesvirus infection, including internalization, endosome sorting, and endosome trafficking. The regulation of cytoskeletons is determined by signals transduced from the interactions between viral glycoproteins and receptors at the cell membrane. KSHV entry regulates actin and microtubule dynamics, in part through the integrin-signaling cascade, to facilitate endocytosis, endosomal sorting and trafficking, and possibly virion surfing. Nevertheless, the exact roles of cytoskeletons in these processes during KSHV primary infection remain unclear. Whether similar events are involved in the RRV entry and trafficking remain to be determined. Further understanding of the involvement of microtubule and actin cytoskeletons, as well as other cellular factors in the entry and trafficking of KSHV, could facilitate the development of inhibitors and vaccines for preventing infection by this oncogenic virus.

## References

[1] Schneider-Schaulies J. (2000). Cellular receptors for viruses: Links to tropism and pathogenesis. J. Gen. Virol..

[2] Marsh M., Helenius A. (2006). Virus entry: Open sesame. Cell.

[3] Greene W., Kuhne K., Ye F., Chen J., Zhou F., Lei X., Gao S.J. (2007). Molecular biology of KSHV in relation to AIDS-associated oncogenesis. Cancer Treat. Res..

[4] Chakraborty S., Veettil M.V., Chandran B. (2012). Kaposi's sarcoma associated herpesvirus entry into target cells. Front. Microbiol..

[5] Chandran B. (2010). Early events in Kaposi's sarcoma-associated herpesvirus infection of target cells. J. Virol..

[6] Lyman M.G., Enquist L.W. (2009). Herpesvirus interactions with the host cytoskeleton. J. Virol..

[7] Van den Broeke C., Favoreel H.W. (2011). Actin' up: Herpesvirus interactions with rho gtpase signaling. Viruses.

[8] Roberts K.L., Baines J.D. (2011). Actin in herpesvirus infection. Viruses.

[9] Janmey P.A. (1998). The cytoskeleton and cell signaling: Component localization and mechanical coupling. Physiol. Rev..

[10] Hahn A.S., Kaufmann J.K., Wies E., Naschberger E., Panteleev-Ivlev J., Schmidt K., Holzer A., Schmidt M., Chen J., Konig S., Ensser A., Myoung J., Brockmeyer N.H., Sturzl M., Fleckenstein B., Neipel F. (2012). The ephrin receptor tyrosine kinase a2 is a cellular receptor for kaposi's sarcoma-associated herpesvirus. Nat. Med..

[11] Gottlieb A.I., Langille B.L., Wong M.K., Kim D.W. (1991). Structure and function of the endothelial cytoskeleton. Lab. Invest..

[12] Collins A., Warrington A., Taylor K.A., Svitkina T. (2011). Structural organization of the actin cytoskeleton at sites of clathrin-mediated endocytosis. Curr. Biol..

[13] Jamora C., Fuchs E. (2002). Intercellular adhesion, signalling and the cytoskeleton. Nat. Cell Biol..

[14] Downing K.H., Nogales E. (1998). Tubulin and microtubule structure. Curr. Opin. Cell Biol..

[15] Dohner K., Sodeik B. (2005). The role of the cytoskeleton during viral infection. Curr. Top. Microbiol. Immunol..

[16] Nogales E. (2000). Structural insights into microtubule function. Annu. Rev. Biochem..

[17] Taylor M.P., Koyuncu O.O., Enquist L.W. (2011). Subversion of the actin cytoskeleton during viral infection. Nat. Rev. Microbiol..

[18] Schmidt A., Hall A. (2002). Guanine nucleotide exchange factors for rho gtpases: Turning on the switch. Genes Dev..

[19] Miller A.L., Bement W.M. (2009). Regulation of cytokinesis by rho gtpase flux. Nat. Cell Biol..

[20] Bishop A.L., Hall A. (2000). Rho gtpases and their effector proteins. Biochem. J..

[21] Anitei M., Hoflack B. (2012). Bridging membrane and cytoskeleton dynamics in the secretory and endocytic pathways. Nat. Cell Biol..

[22] de Forges H., Bouissou A., Perez F. (2012). Interplay between microtubule dynamics and intracellular organization. Int. J. Biochem. Cell Biol..

[23] Westermann S., Weber K. (2003). Post-translational modifications regulate microtubule function. Nat. Rev. Mol. Cell Biol..

[24] Wloga D., Gaertig J. (2010). Post-translational modifications of microtubules. J. Cell Sci..

[25] Dohner K., Nagel C.H., Sodeik B. (2005). Viral stop-and-go along microtubules: Taking a ride with dynein and kinesins. Trends Microbiol..

[26] Gee M.A., Heuser J.E., Vallee R.B. (1997). An extended microtubule-binding structure within the dynein motor domain. Nature.

[27] Howard J., Hudspeth A.J., Vale R.D. (1989). Movement of microtubules by single kinesin molecules. Nature.

[28] Kon T., Nishiura M., Ohkura R., Toyoshima Y.Y., Sutoh K. (2004). Distinct functions of nucleotide-binding/hydrolysis sites in the four aaa modules of cytoplasmic dynein. Biochemistry.

[29] Amorim M.J., Bruce E.A., Read E.K., Foeglein A., Mahen R., Stuart A.D., Digard P. (2011). A rab11- and microtubule-dependent mechanism for cytoplasmic transport of influenza a virus viral rna. J. Virol..

[30] Chambers R., Takimoto T. (2010). Trafficking of sendai virus nucleocapsids is mediated by intracellular vesicles. PLoS One.

[31] Diefenbach R.J., Miranda-Saksena M., Douglas M.W., Cunningham A.L. (2008). Transport and egress of herpes simplex virus in neurons. Rev. Med. Virol..

[32] Lehmann M., Milev M.P., Abrahamyan L., Yao X.J., Pante N., Mouland A.J. (2009). Intracellular transport of human immunodeficiency virus type 1 genomic rna and viral production are dependent on dynein motor function and late endosome positioning. J. Biol. Chem..

[33] Leopold P.L., Kreitzer G., Miyazawa N., Rempel S., Pfister K.K., Rodriguez-Boulan E., Crystal R.G. (2000). Dynein- and microtubule-mediated translocation of adenovirus serotype 5 occurs after endosomal lysis. Hum. Gene Ther..

[34] Ogawa-Goto K., Tanaka K., Gibson W., Moriishi E., Miura Y., Kurata T., Irie S., Sata T. (2003). Microtubule network facilitates nuclear targeting of human cytomegalovirus capsid. J. Virol..

[35] Sodeik B., Ebersold M.W., Helenius A. (1997). Microtubule-mediated transport of incoming herpes simplex virus 1 capsids to the nucleus. J. Cell Biol..

[36] Su Y., Qiao W., Guo T., Tan J., Li Z., Chen Y., Li X., Li Y., Zhou J., Chen Q. (2010). Microtubule-dependent retrograde transport of bovine immunodeficiency virus. Cell. Microbiol..

[37] Suomalainen M., Nakano M.Y., Keller S., Boucke K., Stidwill R.P., Greber U.F. (1999). Microtubule-dependent plus- and minus end-directed motilities are competing processes for nuclear targeting of adenovirus. J. Cell Biol..

[38] Salmikangas P., van der Ven P.F., Lalowski M., Taivainen A., Zhao F., Suila H., Schroder R., Lappalainen P., Furst D.O., Carpen O. (2003). Myotilin, the limb-girdle muscular dystrophy 1a (LGMD1A) protein, cross-links actin filaments and controls sarcomere assembly. Hum. Mol. Genet..

[39] Dominguez R., Holmes K.C. (2011). Actin structure and function. Annu. Rev. Biophys..

[40] Welch M.D., Mullins R.D. (2002). Cellular control of actin nucleation. Annu. Rev. Cell Dev. Biol..

[41] Gill M.B., Edgar R., May J.S., Stevenson P.G. (2008). A gamma-herpesvirus glycoprotein complex manipulates actin to promote viral spread. PLoS One.

[42] Olson E.N., Nordheim A. (2010). Linking actin dynamics and gene transcription to drive cellular motile functions. Nat. Rev. Mol. Cell. Biol..

[43] Bloemink M.J., Geeves M.A. (2011). Shaking the myosin family tree: Biochemical kinetics defines four types of myosin motor. Semin. Cell Dev. Biol..

[44] Sweeney H.L., Houdusse A. (2010). Structural and functional insights into the myosin motor mechanism. Annu. Rev. Biophys..

[45] Hartman M.A., Finan D., Sivaramakrishnan S., Spudich J.A. (2011). Principles of unconventional myosin function and targeting. Annu. Rev. Cell Dev. Biol..

[46] Cramer L.P. (2000). Myosin vi: Roles for a minus end-directed actin motor in cells. J. Cell. Biol..

[47] Greber U.F., Way M. (2006). A superhighway to virus infection. Cell.

[48] Schelhaas M., Shah B., Holzer M., Blattmann P., Kuhling L., Day P.M., Schiller J.T., Helenius A. (2012). Entry of human papillomavirus type 16 by actin-dependent, clathrin- and lipid raft-independent endocytosis. PLoS Pathog..

[49] Li E., Stupack D., Bokoch G.M., Nemerow G.R. (1998). Adenovirus endocytosis requires actin cytoskeleton reorganization mediated by rho family gtpases. J. Virol..

[50] Goldman R.D., Khuon S., Chou Y.H., Opal P., Steinert P.M. (1996). The function of intermediate filaments in cell shape and cytoskeletal integrity. J. Cell. Biol..

[51] Herrmann H., Aebi U. (2000). Intermediate filaments and their associates: Multi-talented structural elements specifying cytoarchitecture and cytodynamics. Curr. Opin. Cell Biol..

[52] Watts N.R., Jones L.N., Cheng N., Wall J.S., Parry D.A., Steven A.C. (2002). Cryo-electron microscopy of trichocyte (hard alpha-keratin) intermediate filaments reveals a low-density core. J. Struct. Biol..

[53] Strelkov S.V., Herrmann H., Aebi U. (2003). Molecular architecture of intermediate filaments. Bioessays.

[54] Roizman B., Sears E., Fields B.N.; Knipe, D.M.; Howley P.M. (1996). Herpes simplex viruses and their replication. Fundamental virology.

[55] Mettenleiter T.C. (2002). Herpesvirus assembly and egress. J. Virol..

[56] Spear P.G., Longnecker R. (2003). Herpesvirus entry: An update. J. Virol..

[57] Longnecker R., Neipel F., Arvin A., Campadelli-Fiume G., Mocarski E., Moore P.S., Roizman B., Whitley R., Yamanishi K. (2007). Introduction to the human gamma-herpesviruses. Human herpesviruses: Biology, therapy, and immunoprophylaxis.

[58] Barton E., Mandal P., Speck S.H. (2011). Pathogenesis and host control of gammaherpesviruses: Lessons from the mouse. Annu. Rev. Immunol..

[59] Bechtel J.T., Liang Y., Hvidding J., Ganem D. (2003). Host range of kaposi's sarcoma-associated herpesvirus in cultured cells. J. Virol..

[60] Sarid R., Gao S.J. (2011). Viruses and human cancer: From detection to causality. Cancer Lett..

[61] Ye F., Lei X., Gao S.J. (2011). Mechanisms of kaposi's sarcoma-associated herpesvirus latency and reactivation. Adv. Virol..

[62] Cai Q., Verma S.C., Lu J., Robertson E.S. (2010). Molecular biology of kaposi's sarcoma-associated herpesvirus and related oncogenesis. Adv. Virus Res..

[63] Speck S.H., Ganem D. (2010). Viral latency and its regulation: Lessons from the gamma-herpesviruses. Cell Host Microbe.

[64] Mesri E.A., Cesarman E., Boshoff C. (2010). Kaposi's sarcoma and its associated herpesvirus. Nat. Rev. Cancer.

[65] Scadden D.T. (2003). Aids-related malignancies. Annu. Rev. Med..

[66] Desrosiers R.C., Sasseville V.G., Czajak S.C., Zhang X., Mansfield K.G., Kaur A., Johnson R.P., Lackner A.A., Jung J.U. (1997). A herpesvirus of rhesus monkeys related to the human kaposi's sarcoma-associated herpesvirus. J. Virol..

[67] Searles R.P., Bergquam E.P., Axthelm M.K., Wong S.W. (1999). Sequence and genomic analysis of a rhesus macaque rhadinovirus with similarity to kaposi's sarcoma-associated herpesvirus/human herpesvirus 8. J. Virol..

[68] Wong S.W., Bergquam E.P., Swanson R.M., Lee F.W., Shiigi S.M., Avery N.A., Fanton J.W., Axthelm M.K. (1999). Induction of b cell hyperplasia in simian immunodeficiency virus-infected rhesus macaques with the simian homologue of kaposi's sarcoma-associated herpesvirus. J. Exp. Med..

[69] Orzechowska B.U., Powers M.F., Sprague J., Li H., Yen B., Searles R.P., Axthelm M.K., Wong S.W. (2008). Rhesus macaque rhadinovirus-associated non-hodgkin lymphoma: Animal model for KSHV-associated malignancies. Blood.

[70] Coutsinos Z., Absi Z., Henin Y., Guillet J.G., Launay O. (2008). Designing an effective aids vaccine: Strategies and current status. Rev. Med. Interne..

[71] Dimitrov D.S. (2004). Virus entry: Molecular mechanisms and biomedical applications. Nat. Rev. Microbiol..

[72] Heldwein E.E., Krummenacher C. (2008). Entry of herpesviruses into mammalian cells. Cell. Mol. Life Sci..

[73] Claesson-Welsh L., Spear P.G. (1986). Oligomerization of herpes simplex virus glycoprotein b. J. Virol..

[74] Pertel P.E. (2002). Human herpesvirus 8 glycoprotein b (gb), gh, and gl can mediate cell fusion. J. Virol..

[75] Russo J.J., Bohenzky R.A., Chien M.C., Chen J., Yan M., Maddalena D., Parry J.P., Peruzzi D., Edelman I.S., Chang Y., Moore P.S. (1996). Nucleotide sequence of the kaposi sarcoma-associated herpesvirus (hhv8). Proc. Natl. Acad. Sci. USA.

[76] Zhu L., Puri V., Chandran B. (1999). Characterization of human herpesvirus-8 k8.1a/b glycoproteins by monoclonal antibodies. Virology.

[77] Shin Y.C., Desrosiers R.C. (2011). Rhesus monkey rhadinovirus orf57 induces gh and gl glycoprotein expression through posttranscriptional accumulation of target mrnas. J. Virol..

[78] Bilello J.P., Morgan J.S., Desrosiers R.C. (2008). Extreme dependence of gh and gl expression on orf57 and association with highly unusual codon usage in rhesus monkey rhadinovirus. J. Virol..

[79] Alexander L., Denekamp L., Knapp A., Auerbach M.R., Damania B., Desrosiers R.C. (2000). The primary sequence of rhesus monkey rhadinovirus isolate 26–95: Sequence similarities to kaposi's sarcoma-associated herpesvirus and rhesus monkey rhadinovirus isolate 17577. J. Virol..

[80] Shin Y.C., Jones L.R., Manrique J., Lauer W., Carville A., Mansfield K.G., Desrosiers R.C. (2010). Glycoprotein gene sequence variation in rhesus monkey rhadinovirus. Virology.

[81] Hutt-Fletcher L.M. (2007). Epstein-barr virus entry. J. Virol..

[82] Connolly S.A., Jackson J.O., Jardetzky T.S., Longnecker R. (2011). Fusing structure and function: A structural view of the herpesvirus entry machinery. Nat. Rev. Microbiol..

[83] Sarrazin S., Lamanna W.C., Esko J.D. (2011). Heparan sulfate proteoglycans. Cold Spring Harb. Perspect. Biol..

[84] Zhu W., Li J., Liang G. (2011). How does cellular heparan sulfate function in viral pathogenicity?. Biomed. Environ. Sci..

[85] Shukla D., Spear P.G. (2001). Herpesviruses and heparan sulfate: An intimate relationship in aid of viral entry. J. Clin. Invest..

[86] WuDunn D., Spear P.G. (1989). Initial interaction of herpes simplex virus with cells is binding to heparan sulfate. J. Virol..

[87] Shieh M.T., WuDunn D., Montgomery R.I., Esko J.D., Spear P.G. (1992). Cell surface receptors for herpes simplex virus are heparan sulfate proteoglycans. J. Cell Biol..

[88] Shukla D., Liu J., Blaiklock P., Shworak N.W., Bai X., Esko J.D., Cohen G.H., Eisenberg R.J., Rosenberg R.D., Spear P.G. (1999). A novel role for 3-o-sulfated heparan sulfate in herpes simplex virus 1 entry. Cell.

[89] O'Donnell C.D., Shukla D. (2008). The importance of heparan sulfate in herpesvirus infection. Virol. Sin..

[90] Akula S.M., Pramod N.P., Wang F.Z., Chandran B. (2001). Human herpesvirus 8 envelope-associated glycoprotein b interacts with heparan sulfate-like moieties. Virology.

[91] Akula S.M., Wang F.Z., Vieira J., Chandran B. (2001). Human herpesvirus 8 interaction with target cells involves heparan sulfate. Virology.

[92] Hahn A., Birkmann A., Wies E., Dorer D., Mahr K., Sturzl M., Titgemeyer F., Neipel F. (2009). Kaposi's sarcoma-associated herpesvirus gh/gl: Glycoprotein export and interaction with cellular receptors. J. Virol..

[93] Wang F.Z., Akula S.M., Pramod N.P., Zeng L., Chandran B. (2001). Human herpesvirus 8 envelope glycoprotein K8.1a interaction with the target cells involves heparan sulfate. J. Virol..

[94] Birkmann A., Mahr K., Ensser A., Yaguboglu S., Titgemeyer F., Fleckenstein B., Neipel F. (2001). Cell surface heparan sulfate is a receptor for human herpesvirus 8 and interacts with envelope glycoprotein K8.1. J. Virol..

[95] Luna R.E., Zhou F., Baghian A., Chouljenko V., Forghani B., Gao S.J., Kousoulas K.G. (2004). Kaposi’s sarcoma-associated herpesvirus glycoprotein K8.1 is dispensable for virus entry. J. Virol..

[96] Zhang W., Zhou F., Greene W., Gao S.J. (2010). Rhesus rhadinovirus infection of rhesus fibroblasts occurs through clathrin-mediated endocytosis. J. Virol..

[97] Grove J., Marsh M. (2011). The cell biology of receptor-mediated virus entry. J. Cell Biol..

[98] Jha N.K., Latinovic O., Martin E., Novitskiy G., Marin M., Miyauchi K., Naughton J., Young J.A., Melikyan G.B. (2011). Imaging single retrovirus entry through alternative receptor isoforms and intermediates of virus-endosome fusion. PLoS Pathog..

[99] Lin G., Simmons G., Pohlmann S., Baribaud F., Ni H., Leslie G.J., Haggarty B.S., Bates P., Weissman D., Hoxie J.A., Doms R.W. (2003). Differential n-linked glycosylation of human immunodeficiency virus and ebola virus envelope glycoproteins modulates interactions with dc-sign and dc-signr. J. Virol..

[100] Alvarez C.P., Lasala F., Carrillo J., Muniz O., Corbi A.L., Delgado R. (2002). C-type lectins dc-sign and l-sign mediate cellular entry by ebola virus in cis and in trans. J. Virol..

[101] Halary F., Amara A., Lortat-Jacob H., Messerle M., Delaunay T., Houles C., Fieschi F., Arenzana-Seisdedos F., Moreau J.F., Dechanet-Merville J. (2002). Human cytomegalovirus binding to dc-sign is required for dendritic cell infection and target cell trans-infection. Immunity.

[102] Tassaneetrithep B., Burgess T.H., Granelli-Piperno A., Trumpfheller C., Finke J., Sun W., Eller M.A., Pattanapanyasat K., Sarasombath S., Birx D.L., Steinman R.M., Schlesinger S., Marovich M.A. (2003). Dc-sign (cd209) mediates dengue virus infection of human dendritic cells. J. Exp. Med..

[103] Kwon D.S., Gregorio G., Bitton N., Hendrickson W.A., Littman D.R. (2002). Dc-sign-mediated internalization of hiv is required for trans-enhancement of t cell infection. Immunity.

[104] Kerur N., Veettil M.V., Sharma-Walia N., Sadagopan S., Bottero V., Paul A.G., Chandran B. (2010). Characterization of entry and infection of monocytic thp-1 cells by Kaposi's sarcoma associated herpesvirus (KSHV): Role of heparan sulfate, dc-sign, integrins and signaling. Virology.

[105] Rappocciolo G., Hensler H.R., Jais M., Reinhart T.A., Pegu A., Jenkins F.J., Rinaldo C.R. (2008). Human herpesvirus 8 infects and replicates in primary cultures of activated b lymphocytes through dc-sign. J. Virol..

[106] Rappocciolo G., Jenkins F.J., Hensler H.R., Piazza P., Jais M., Borowski L., Watkins S.C., Rinaldo C.R. (2006). Dc-sign is a receptor for human herpesvirus 8 on dendritic cells and macrophages. J. Immunol..

[107] Jarousse N., Chandran B., Coscoy L. (2008). Lack of heparan sulfate expression in b-cell lines: Implications for kaposi's sarcoma-associated herpesvirus and murine gammaherpesvirus 68 infections. J. Virol..

[108] Ye F., Kim C., Ginsberg M.H. (2012). Reconstruction of integrin activation. Blood.

[109] Giancotti F.G., Ruoslahti E. (1999). Integrin signaling. Science.

[110] Stewart P.L., Nemerow G.R. (2007). Cell integrins: Commonly used receptors for diverse viral pathogens. Trends Microbiol..

[111] Akula S.M., Pramod N.P., Wang F.Z., Chandran B. (2002). Integrin alpha3beta1 (cd 49c/29) is a cellular receptor for Kaposi's sarcoma-associated herpesvirus (KSHV/HHV-8) entry into the target cells. Cell.

[112] Plow E.F., Haas T.A., Zhang L., Loftus J., Smith J.W. (2000). Ligand binding to integrins. J. Biol. Chem..

[113] Humphries J.D., Byron A., Humphries M.J. (2006). Integrin ligands at a glance. J. Cell Sci..

[114] Wang F.Z., Akula S.M., Sharma-Walia N., Zeng L., Chandran B. (2003). Human herpesvirus 8 envelope glycoprotein b mediates cell adhesion via its rgd sequence. J. Virol..

[115] Veettil M.V., Sadagopan S., Sharma-Walia N., Wang F.Z., Raghu H., Varga L., Chandran B. (2008). Kaposi's sarcoma-associated herpesvirus forms a multimolecular complex of integrins (alphavbeta5, alphavbeta3, and alpha3beta1) and cd98-xct during infection of human dermal microvascular endothelial cells, and cd98-xct is essential for the postentry stage of infection. J. Virol..

[116] Inoue N., Winter J., Lal R.B., Offermann M.K., Koyano S. (2003). Characterization of entry mechanisms of human herpesvirus 8 by using an rta-dependent reporter cell line. J. Virol..

[117] Kaleeba J.A., Berger E.A. (2006). Broad target cell selectivity of kaposi's sarcoma-associated herpesvirus glycoprotein-mediated cell fusion and virion entry. Virology.

[118] Garrigues H.J., Rubinchikova Y.E., Dipersio C.M., Rose T.M. (2008). Integrin alphavbeta3 binds to the rgd motif of glycoprotein b of kaposi's sarcoma-associated herpesvirus and functions as an rgd-dependent entry receptor. J. Virol..

[119] Rahman S., Aitken A., Flynn G., Formstone C., Savidge G.F. (1998). Modulation of rgd sequence motifs regulates disintegrin recognition of alphaiib beta3 and alpha5 beta1 integrin complexes. Replacement of elegantin alanine-50 with proline, n-terminal to the rgd sequence, diminishes recognition of the alpha5 beta1 complex with restoration induced by mn2+ cation. Biochem. J..

[120] Miao H., Li D.Q., Mukherjee A., Guo H., Petty A., Cutter J., Basilion J.P., Sedor J., Wu J., Danielpour D., Sloan A.E., Cohen M.L., Wang B. (2009). Epha2 mediates ligand-dependent inhibition and ligand-independent promotion of cell migration and invasion via a reciprocal regulatory loop with akt. Cancer Cell.

[121] Pasquale E.B. (2008). Eph-ephrin bidirectional signaling in physiology and disease. Cell.

[122] Nakamoto M., Bergemann A.D. (2002). Diverse roles for the eph family of receptor tyrosine kinases in carcinogenesis. Microsc. Res. Tech..

[123] Aasheim H.C., Munthe E., Funderud S., Smeland E.B., Beiske K., Logtenberg T. (2000). A splice variant of human ephrin-a4 encodes a soluble molecule that is secreted by activated human b lymphocytes. Blood.

[124] Naranatt P.P., Akula S.M., Chandran B. (2002). Characterization of gamma2-human herpesvirus-8 glycoproteins gh and gl. Arch. Virol..

[125] Chakraborty S., Veettil M.V., Bottero V., Chandran B. (2012). Kaposi's sarcoma-associated herpesvirus interacts with ephrina2 receptor to amplify signaling essential for productive infection. Proc. Natl. Acad. Sci. USA.

[126] He M., Zhang W., Bakken T., Schutten M., Toth Z., Jung J.U., Gill P., Cannon M., Gao S.J. (2012). Cancer angiogenesis induced by kaposi's sarcoma-associated herpesvirus is mediated by ezh2. Cancer Res..

[127] Tsuchiya S., Kobayashi Y., Goto Y., Okumura H., Nakae S., Konno T., Tada K. (1982). Induction of maturation in cultured human monocytic leukemia cells by a phorbol diester. Cancer Res..

[128] Lo M., Wang Y.Z., Gout P.W. (2008). The x(c)- cystine/glutamate antiporter: A potential target for therapy of cancer and other diseases. J. Cell Physiol..

[129] Kaleeba J.A., Berger E.A. (2006). Kaposi's sarcoma-associated herpesvirus fusion-entry receptor: Cystine transporter xCT. Science.

[130] Sieczkarski S.B., Whittaker G.R. (2002). Dissecting virus entry via endocytosis. J. Gen. Virol..

[131] Nemerow G.R., Cooper N.R. (1984). Early events in the infection of human b lymphocytes by epstein-barr virus: The internalization process. Virology.

[132] Miller N., Hutt-Fletcher L.M. (1992). Epstein-barr virus enters b cells and epithelial cells by different routes. J. Virol..

[133] Szakonyi G., Klein M.G., Hannan J.P., Young K.A., Ma R.Z., Asokan R., Holers V.M., Chen X.S. (2006). Structure of the epstein-barr virus major envelope glycoprotein. Nat. Struct. Mol. Biol..

[134] Seigneurin J.M., Vuillaume M., Lenoir G., De-The G. (1977). Replication of epstein-barr virus: Ultrastructural and immunofluorescent studies of p3hr1-superinfected raji cells. J. Virol..

[135] Akula S.M., Naranatt P.P., Walia N.S., Wang F.Z., Fegley B., Chandran B. (2003). Kaposi's sarcoma-associated herpesvirus (human herpesvirus 8) infection of human fibroblast cells occurs through endocytosis. J. Virol..

[136] Greene W., Gao S.J. (2009). Actin dynamics regulate multiple endosomal steps during kaposi's sarcoma-associated herpesvirus entry and trafficking in endothelial cells. PLoS Pathog..

[137] Raghu H., Sharma-Walia N., Veettil M.V., Sadagopan S., Chandran B. (2009). Kaposi's sarcoma-associated herpesvirus utilizes an actin polymerization-dependent macropinocytic pathway to enter human dermal microvascular endothelial and human umbilical vein endothelial cells. J. Virol..

[138] Brandenburg B., Zhuang X. (2007). Virus trafficking - learning from single-virus tracking. Nat. Rev. Microbiol..

[139] Smith G.A., Enquist L.W. (2002). Break ins and break outs: Viral interactions with the cytoskeleton of mammalian cells. Annu. Rev. Cell Dev. Biol..

[140] Greene W., Zhang W., He M., Witt C., Ye F., Gao S.J. (2012). The ubiquitin/proteasome system mediates entry and endosomal trafficking of kaposi's sarcoma-associated herpesvirus in endothelial cells. PLoS Pathog..

[141] Zhang W., Greene W., Gao S.J. (2012). Microtubule- and dynein-dependent nuclear trafficking of rhesus rhadinovirus in rhesus fibroblasts. J. Virol..

[142] Benmerah A., Bayrou M., Cerf-Bensussan N., Dautry-Varsat A. (1999). Inhibition of clathrin-coated pit assembly by an eps15 mutant. J. Cell Sci..

[143] Benmerah A., Lamaze C., Begue B., Schmid S.L., Dautry-Varsat A., Cerf-Bensussan N. (1998). Ap-2/eps15 interaction is required for receptor-mediated endocytosis. J. Cell Biol..

[144] Naranatt P.P., Krishnan H.H., Smith M.S., Chandran B. (2005). Kaposi's sarcoma-associated herpesvirus modulates microtubule dynamics via rhoa-gtp-diaphanous 2 signaling and utilizes the dynein motors to deliver its DNA to the nucleus. J. Virol..

[145] Burkhardt J.K., Echeverri C.J., Nilsson T., Vallee R.B. (1997). Overexpression of the dynamitin (p50) subunit of the dynactin complex disrupts dynein-dependent maintenance of membrane organelle distribution. J. Cell Biol..

[146] Burckhardt C.J., Suomalainen M., Schoenenberger P., Boucke K., Hemmi S., Greber U.F. (2011). Drifting motions of the adenovirus receptor car and immobile integrins initiate virus uncoating and membrane lytic protein exposure. Cell Host Microbe.

[147] Medeiros N.A., Burnette D.T., Forscher P. (2006). Myosin ii functions in actin-bundle turnover in neuronal growth cones. Nat. Cell Biol..

[148] Lehmann M.J., Sherer N.M., Marks C.B., Pypaert M., Mothes W. (2005). Actin- and myosin-driven movement of viruses along filopodia precedes their entry into cells. J. Cell Biol..

[149] Arii J., Goto H., Suenaga T., Oyama M., Kozuka-Hata H., Imai T., Minowa A., Akashi H., Arase H., Kawaoka Y., Kawaguchi Y. (2010). Non-muscle myosin iia is a functional entry receptor for herpes simplex virus-1. Nature.

[150] Clement C., Tiwari V., Scanlan P.M., Valyi-Nagy T., Yue B.Y., Shukla D. (2006). A novel role for phagocytosis-like uptake in herpes simplex virus entry. J. Cell Biol..

[151] Petermann P., Haase I., Knebel-Morsdorf D. (2009). Impact of rac1 and cdc42 signaling during early herpes simplex virus type 1 infection of keratinocytes. J. Virol..

[152] Hoppe S., Schelhaas M., Jaeger V., Liebig T., Petermann P., Knebel-Morsdorf D. (2006). Early herpes simplex virus type 1 infection is dependent on regulated rac1/cdc42 signalling in epithelial mdckii cells. J. Gen. Virol..

[153] Wittels M., Spear P.G. (1991). Penetration of cells by herpes simplex virus does not require a low ph-dependent endocytic pathway. Virus Res..

[154] Valencia S.M., Hutt-Fletcher L.M. (2012). Important but differential roles for actin in trafficking of epstein-barr virus in b cells and epithelial cells. J. Virol..

[155] Jones N.L., Lewis J.C., Kilpatrick B.A. (1986). Cytoskeletal disruption during human cytomegalovirus infection of human lung fibroblasts. Eur. J. Cell Biol..

[156] Krishnan H.H., Sharma-Walia N., Streblow D.N., Naranatt P.P., Chandran B. (2006). Focal adhesion kinase is critical for entry of kaposi's sarcoma-associated herpesvirus into target cells. J. Virol..

[157] Chrzanowska-Wodnicka M., Burridge K. (1996). Rho-stimulated contractility drives the formation of stress fibers and focal adhesions. J. Cell Biol..

[158] Naranatt P.P., Akula S.M., Zien C.A., Krishnan H.H., Chandran B. (2003). Kaposi's sarcoma-associated herpesvirus induces the phosphatidylinositol 3-kinase-pkc-zeta-mek-erk signaling pathway in target cells early during infection: Implications for infectivity. J. Virol..

[159] Sharma-Walia N., Naranatt P.P., Krishnan H.H., Zeng L., Chandran B. (2004). Kaposi's sarcoma-associated herpesvirus/human herpesvirus 8 envelope glycoprotein gb induces the integrin-dependent focal adhesion kinase-src-phosphatidylinositol 3-kinase-rho gtpase signal pathways and cytoskeletal rearrangements. J. Virol..

[160] Veettil M.V., Sharma-Walia N., Sadagopan S., Raghu H., Sivakumar R., Naranatt P.P., Chandran B. (2006). Rhoa-gtpase facilitates entry of kaposi's sarcoma-associated herpesvirus into adherent target cells in a src-dependent manner. J. Virol..

[161] Wilde A., Beattie E.C., Lem L., Riethof D.A., Liu S.H., Mobley W.C., Soriano P., Brodsky F.M. (1999). Egf receptor signaling stimulates src kinase phosphorylation of clathrin, influencing clathrin redistribution and egf uptake. Cell.

[162] Galletta B.J., Mooren O.L., Cooper J.A. (2010). Actin dynamics and endocytosis in yeast and mammals. Curr. Opin. Biotechnol..

[163] Schmid S.L. (1997). Clathrin-coated vesicle formation and protein sorting: An integrated process. Annu. Rev. Biochem..

[164] Brodsky F.M., Chen C.Y., Knuehl C., Towler M.C., Wakeham D.E. (2001). Biological basket weaving: Formation and function of clathrin-coated vesicles. Annu. Rev. Cell Dev. Biol..

[165] Yarar D., Waterman-Storer C.M., Schmid S.L. (2005). A dynamic actin cytoskeleton functions at multiple stages of clathrin-mediated endocytosis. Mol. Biol. Cell.

[166] Schmidt M.H., Dikic I. (2005). The cbl interactome and its functions. Nat. Rev. Mol. Cell. Biol..

[167] Valiya Veettil M., Sadagopan S., Kerur N., Chakraborty S., Chandran B. (2010). Interaction of c-cbl with myosin iia regulates bleb associated macropinocytosis of kaposi's sarcoma-associated herpesvirus. PLoS Pathog..

[168] Mercer J., Helenius A. (2009). Virus entry by macropinocytosis. Nat. Cell Biol..

[169] Chakraborty S., ValiyaVeettil M., Sadagopan S., Paudel N., Chandran B. (2011). C-cbl-mediated selective virus-receptor translocations into lipid rafts regulate productive kaposi's sarcoma-associated herpesvirus infection in endothelial cells. J. Virol..

[170] Ohashi E., Tanabe K., Henmi Y., Mesaki K., Kobayashi Y., Takei K. (2011). Receptor sorting within endosomal trafficking pathway is facilitated by dynamic actin filaments. PLoS One.

[171] Hurley J.H., Stenmark H. (2011). Molecular mechanisms of ubiquitin-dependent membrane traffic. Annu. Rev. Biophys..

[172] Hicke L. (2001). Protein regulation by monoubiquitin. Nat. Rev. Mol. Cell. Biol..

[173] Pan H., Xie J., Ye F., Gao S.J. (2006). Modulation of Kaposi's sarcoma-associated herpesvirus infection and replication by mek/erk, jnk, and p38 multiple mitogen-activated protein kinase pathways during primary infection. J. Virol..

[174] Sharma-Walia N., Krishnan H.H., Naranatt P.P., Zeng L., Smith M.S., Chandran B. (2005). Erk1/2 and mek1/2 induced by Kaposi's sarcoma-associated herpesvirus (human herpesvirus 8) early during infection of target cells are essential for expression of viral genes and for establishment of infection. J. Virol..

[175] Xie J., Pan H., Yoo S., Gao S.J. (2005). Kaposi's sarcoma-associated herpesvirus induction of ap-1 and interleukin 6 during primary infection mediated by multiple mitogen-activated protein kinase pathways. J. Virol..

[176] Qian L.W., Xie J., Ye F., Gao S.J. (2007). Kaposi's sarcoma-associated herpesvirus infection promotes invasion of primary human umbilical vein endothelial cells by inducing matrix metalloproteinases. J. Virol..

[177] Ye F.C., Blackbourn D.J., Mengel M., Xie J.P., Qian L.W., Greene W., Yeh I.T., Graham D., Gao S.J. (2007). Kaposi's sarcoma-associated herpesvirus promotes angiogenesis by inducing angiopoietin-2 expression via ap-1 and ets1. J. Virol..

[178] Qian L.W., Greene W., Ye F., Gao S.J. (2008). Kaposi's sarcoma-associated herpesvirus disrupts adherens junctions and increases endothelial permeability by inducing degradation of ve-cadherin. J. Virol..

[179] Yoon M., Spear P.G. (2002). Disruption of adherens junctions liberates nectin-1 to serve as receptor for herpes simplex virus and pseudorabies virus entry. J. Virol..

